# In Vitro and In Vivo Synergism of Fosfomycin in Combination with Meropenem or Polymyxin B against KPC-2-Producing *Klebsiella pneumoniae* Clinical Isolates

**DOI:** 10.3390/antibiotics12020237

**Published:** 2023-01-23

**Authors:** Aghata Cardoso da Silva Ribeiro, Yohanna Carvalho dos Santos Aoun Chikhani, Tiago Barcelos Valiatti, André Valêncio, Mariana Neri Lucas Kurihara, Fernanda Fernandes Santos, Luciene Andrade da Rocha Minarini, Ana Cristina Gales

**Affiliations:** 1 Laboratório Alerta, Division of Infectious Diseases, Department of Internal Medicine, Escola Paulista de Medicina, Universidade Federal de São Paulo—UNIFESP, São Paulo 04039-032, Brazil; 2 Laboratório Multidisciplinar em Saúde e Meio Ambiente, Departamento de Ciências Farmacêuticas, Instituto de Ciências Ambientais, Químicas e Farmacêuticas, Universidade Federal de São Paulo—UNIFESP, São Paulo 04039-032, Brazil

**Keywords:** fosfomycin, synergism, *Klebsiella pneumoniae*, antimicrobial resistance, *Galleria mellonella*, biofilm

## Abstract

Fosfomycin disodium is a potential therapeutic option to manage difficult-to-treat infections, especially when combined with other antimicrobials. In this study, we evaluated the activity of fosfomycin in combination with meropenem or polymyxin B against contemporaneous KPC-2-producing *K. pneumoniae* clinical isolates (KPC-KPN). Synergistic activity was assessed by checkerboard (CKA) and time–kill (TKA) assays. TKA was performed using serum peak and trough concentrations. The activity of these combinations was also assessed in the *Galleria mellonella* model. Biofilm disruption was assessed by the microtiter plate technique. CKA resulted in an 8- to 2048-fold decrease in meropenem MIC, restoring meropenem activity for 82.4% of the isolates when combined with fosfomycin. For the fosfomycin + polymyxin B combination, a 2- to 128-fold reduction in polymyxin B MIC was achieved, restoring polymyxin B activity for 47% of the isolates. TKA resulted in the synergism of fosfomycin + meropenem (3.0–6.7 log_10_ CFU/mL decrease) and fosfomycin + polymyxin B (6.0–6.2 log_10_ CFU/mL decrease) at peak concentrations. All larvae treated with fosfomycin + meropenem survived. Larvae survival rate was higher with fosfomycin monotherapy (95%) than that observed for fosfomycin + polymyxin B (75%) (*p*-value < 0.0001). Finally, a higher biofilm disruption was observed under exposure to fosfomycin + polymyxin B (2.4–3.4-fold reduction). In summary, we observed a synergistic effect of fosfomycin + meropenem and fosfomycin + polymyxin B combinations, in vitro and in vivo, against KPC-KPN, as well as biofilm disruption.

## 1. Introduction

Infections caused by carbapenem-resistant *Klebsiella pneumoniae* (CRKP) are considered difficult-to-treat infections because treatment options are scarce. This fact led to difficulties in managing these infections, evidencing the need for treatment alternatives [1,2]. The new β-lactamase inhibitor-β-lactam combinations (IBL-BL) currently represent the main therapeutic options for the treatment of KPC-KPN infections. However, these agents are not widely available in low- and middle-income countries (LMICS) due to registration delays and costs [3]. In addition, increasing IBL-BL resistance rates have been reported [4,5,6].

In this sense, old antimicrobial agents such as polymyxins and fosfomycin have gained attention. Polymyxins have been used as last resort therapy, and fosfomycin has emerged as a potential option in the treatment of systemic infections [7,8,9]. Discovered more than 40 years ago, fosfomycin is a bactericidal drug with activity against Gram-positive and Gram-negative bacteria, including multidrug-resistant (MDR) isolates. Moreover, cross-resistance with other antibacterial agents is very uncommon since fosfomycin has a unique chemical structure and mechanism of action, inhibiting peptidoglycan synthesis at an earlier stage compared to β-lactams. Importantly, this drug demonstrates a favorable efficacy and safety profile, and it can be prescribed even for pregnant patients [10,11].

Fosfomycin trometamol is commonly used to treat urinary tract infections (UTIs), but the intravenous use of fosfomycin disodium has been prescribed for the treatment of MDR infections, usually in combination due to the risk of resistance selection [12]. Recently, the IDSA recommended against the use of fosfomycin to treat systemic infections caused by CRKPN, especially because this species harbors *fosA* in their chromosome, which codifies a glutathione transferase capable of inactivating the fosfomycin molecule. Besides that, the intravenous formulation of fosfomycin is not available in the United States of America [13]. Combination therapy aims to maximize bacterial killing and minimize the emergence of MDR bacteria. Fosfomycin combination therapy has shown to be superior to fosfomycin monotherapy, as reported by Al-Quraini and collaborators, who showed an important fosfomycin minimum inhibitory concentration (MIC) decrease in *K. pneumoniae* [14]. Nevertheless, distinct studies report conflicting results for combinations with fosfomycin. Depending on the isolate’s β-lactamase content, the synergistic activity of the combination can vary, as shown by Wistrand-Yuen et al. These authors reported synergistic activity of fosfomycin + polymyxin B for 60% of KPC-, NDM-, and OXA-48-KPN producers [15]. In contrast, Bakthavatchalam et al. observed that the same combination exhibited 16% synergistic activity against a collection of NDM- and OXA-48-KPN producers [16].

In addition, there is also a discussion about which antimicrobial classes could represent the best combinations to achieve the highest synergism activity. Ideally, antimicrobials possessing distinct mechanisms of action would act at different stages of bacterial metabolism favoring bacterial killing and avoiding the selection and growth of resistant isolates. Fosfomycin and meropenem act to inhibit the bacterial cell wall synthesis, whereas in different stages: fosfomycin inhibits the MurA enzyme, blocking the murein monomers (peptidoglycan precursors) production, while meropenem, a β-lactam, acts binding transpeptidases enzymes, impairing the cross-linking of peptidoglycan subunits, blocking the cell wall synthesis [17,18]. Polymyxin, however, binds to lipopolysaccharide and phospholipids in the outer cell membrane in Gram-negative bacteria, destabilizing it [19].

Although *Galleria mellonella* larvae have a small size, it has been employed to assess pathogen virulence and the effectiveness of antimicrobial combinations because their innate immunity can recognize foreign conserved molecules on the bacteria surface and humoral (soluble) components via pattern recognition receptors [20]. In addition, *G. mellonella* has impressive evolutionary conservation close to mammals [21] and has a fast life cycle and ethical acceptance, making it an attractive low-cost model for testing survival rates [22].

Therefore, in this study, we evaluated the in vitro activity of fosfomycin alone and in combination with meropenem or polymyxin B against previously genetically characterized KPC-KPN isolates using checkerboard and time–kill assays. By testing these combinations, we observed a relevant decrease in meropenem and polymyxin B minimal inhibitory concentrations (MICs) among the tested isolates. Furthermore, we performed an in vivo assay using the *Galleria mellonella* survival model to evaluate the in vivo efficacy of these combinations. We also analyzed the effect of these antimicrobial combinations on biofilm disruption using the microtiter plate technique. Based on our results, we hypothesize that fosfomycin in combination with meropenem or polymyxin B could be a potential therapeutic option to treat difficult-to-treat infections caused by KPC-2-producing *K. pneumoniae*.

## 2. Results

### 2.1. Antimicrobial Susceptibility Profile of KPC-2-Producing K. pneumoniae Isolates

The antimicrobial susceptibility profile displayed by each isolate is shown in Table S3. Among the 17 KPC-KPN analyzed, only four were resistant to fosfomycin (MICs, 64–256 mg/L). Most isolates were susceptible to fosfomycin (82.3%) and resistant to meropenem (88.2%; MIC, 4 to 128 mg/L) and polymyxin B (70.6%; MIC, 1 to 64 mg/L). All KPC-KPN were resistant to aztreonam (MIC, 64 to >64 mg/L), ertapenem (MIC, 16 to >256 mg/L), gentamycin (MIC, 4 to >64 mg/L), ciprofloxacin, (MIC, >64 mg/L), levofloxacin (MIC, 16 to >64 mg/L), ceftazidime (MIC,128 to >256 mg/L), ceftriaxone (MIC, 256 to >256 mg/L), and cefepime (MIC,128 to >256 mg/L). The lowest resistance rate was observed for fosfomycin (23.5%), followed by amikacin (64.7%; MIC, 4 to >64 mg/L), polymyxin B (70.6%), meropenem (88.2%), and imipenem (94.1%, MIC, 4 to 256 mg/L).

### 2.2. In Vitro Synergism Assays

CKA test showed a synergistic effect of fosfomycin + meropenem against 58.8% of the isolates (10/17) and partial synergism against 29.4% (5/17). We observed a decrease in MICs varying from 2- to 16-fold and 8- to 2048-fold for fosfomycin and meropenem, respectively. In addition, we observed restored meropenem activity for 14 isolates (82.4%), with the remaining three isolates changing their susceptibility category from resistant (R) to susceptible, increasing the exposure (I). The meropenem MIC_50_ and MIC_90_ varied from 64 mg/L/128 mg/L to 0.25 mg/L/4 mg/L when combined with fosfomycin, while the fosfomycin MIC_50_ and MIC_90_ varied from 16 mg/L to 256 mg/L, respectively, to 8 mg/L and 32 mg/L (Table 1).

The combination of fosfomycin with polymyxin B achieved a synergism rate of 47.1% (8/17) and partial synergism of 29.4% (5/17). The MICs decreases were from two- to eight-fold for fosfomycin and from two- to 128-fold for polymyxin B. The restored polymyxin B activity was achieved for eight isolates, and for a single isolate, its susceptibility category changed from resistant (R) to susceptible, increasing the exposure (I). The polymyxin B MIC_50_ and MIC_90_ varied from 8 mg/L and 64 mg/L to 0.5 mg/L and 8 mg/L for polymyxin B in combination; for fosfomycin, MIC_50_ and MIC_90_ in combination were 8 mg/L and 64 mg/L, respectively (Table 1).

In general, we observed a higher rate of synergism between fosfomycin + meropenem (58.8%). For those isolates harboring *bla*_KPC-2_ and ESBL genes (*bla*_CTX-M-15_ and/or *bla*_CTX-M-14_ and/or *bla*_OXA-1_), the synergy rates obtained for the two combinations (fosfomycin + meropenem and fosfomycin + polymyxin B) were equal. For the four isolates, not co-harboring ESBL genes, the most active combination was fosfomycin + meropenem (Table 2).

Furthermore, no differences in the activity of both combinations were found when analyzing the polymyxin resistance determinants *mgrB, crrB, pmrA, pmrB,* and *pmrC*. We observed that mutations in these genes did not influence the activity of the determined antimicrobial combination.

Regarding the TKA, six representative isolates of KPC-2-producing *K. pneumoniae* isolates (HSP80, HSP84, HSP06, HSP29, HSP83, and P05) presenting synergism or partial synergism results in CKA, harboring different resistance genes, presenting different phenotypes and belonging to distinct STs were submitted to TKA. The bacterial colony counting at 24 h compared with the initial inoculum (t = 0) in the presence of fosfomycin, meropenem, and polymyxin B, and the respective combinations are shown in Table 3. In general, we could observe a mean decrease of 0.59 log_10_ CFU/mL for fosfomycin at peak concentration and a 3.07 log_10_ CFU/mL mean increase at trough concentration. For meropenem, we observed a mean increase of 1.57 log_10_ CFU/mL at peak and 2.85 log_10_ CFU/mL at trough concentration. Polymyxin B presented a mean decrease of −1.46 log_10_ CFU/mL at peak and 2.31 log_10_ CFU/mL increase at trough concentration (Table S1).

Peak fosfomycin concentration resulted in a mean decrease in CFU counting of 3.32 log_10_ CFU/mL among the tested isolates at 3 h. After this time, it was observed regrowth for all isolates (Table S1). For meropenem, peak concentration provided a reduction in CFU counting (mean of 0.79 CFU/mL) for four isolates at 3 h, with an observed increase from 6 h onward. Just a single isolate (HSP29) did not show growth at 24 h (Table S1). Peak polymyxin B caused a mean 3.63 log_10_ CFU/mL reduction at 6 h, being observed regrowth for five isolates. Although trough concentration decreased the colonies counting for three isolates at 3 h (mean of 0.49 log_10_ CFU/mL), regrowth was observed after this period (Table S1).

**Figure 1:**
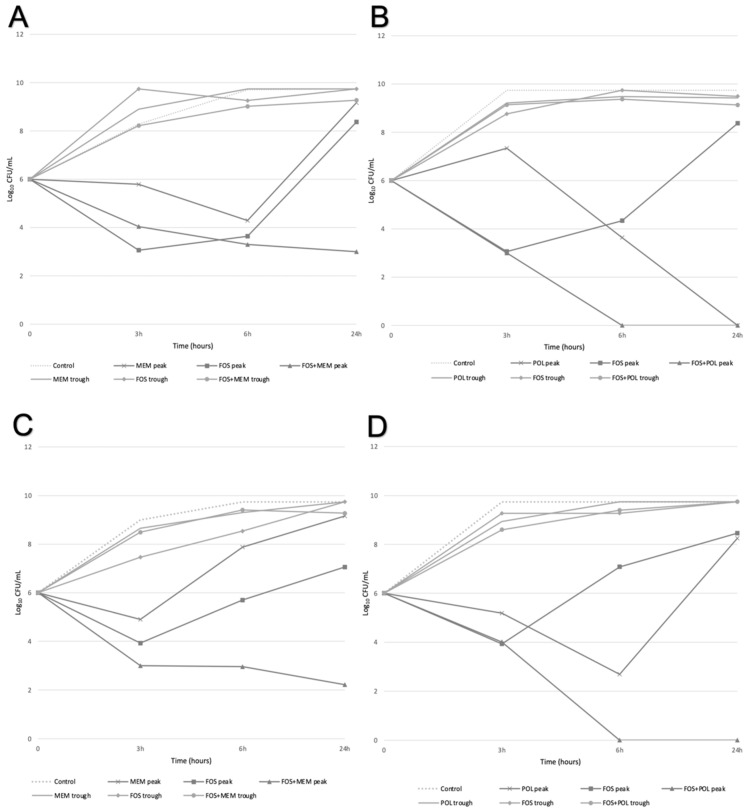
Representative time–kill curves under exposure to peak and trough concentrations of antimicrobials. This figure shows the time–kill curves of two representative isolates (HSP84 and HSP80) exposed to fosfomycin alone, meropenem alone, polymyxin B alone, fosfomycin + meropenem combination, and fosfomycin + polymyxin B combination. (**A**) Time–kill curves for isolate HSP84 under fosfomycin alone, meropenem alone, and fosfomycin + meropenem combination; (**B**) time–kill curves for isolate HSP84 under fosfomycin alone, polymyxin B alone, and fosfomycin + polymyxin B combination; (**C**) time–kill curves for isolate HSP80 under fosfomycin alone, meropenem alone and fosfomycin + meropenem combination; (**D**) time–kill curves for isolate HSP80 under fosfomycin alone, polymyxin B alone and fosfomycin + polymyxin B combination.

The antimicrobial combination of fosfomycin + meropenem at peak concentration was synergic against four isolates at 24 h (Table 3); these isolates also presented a decrease of 2.70–6.67 log_10_ CFU/mL at 6 h (Table S1). For the combination fosfomycin+ polymyxin B, we observed synergism against five isolates (four at peak and one at trough concentrations) at 24 h (Table 3). At peak concentrations, it was achieved a decreased range of 2.2–6.14 log_10_ CFU/mL at 3 h (Table S1). In addition, it was observed a synergistic effect of both combinations at peak concentration against three isolates (HSP80, HSP83, P05) (Table 3). The CKA and TKA results are summarized in Table 4 and Figure 1 displays the time-kill curves of two representatives isolates.

### 2.3. In Vivo Synergism Assay

The toxicity of antimicrobials alone or in combination, in trough and peak concentrations, was tested against 10 larvae each. No evidence of toxicity was noticed because all larvae remained alive five days after.

**Figure 2:**
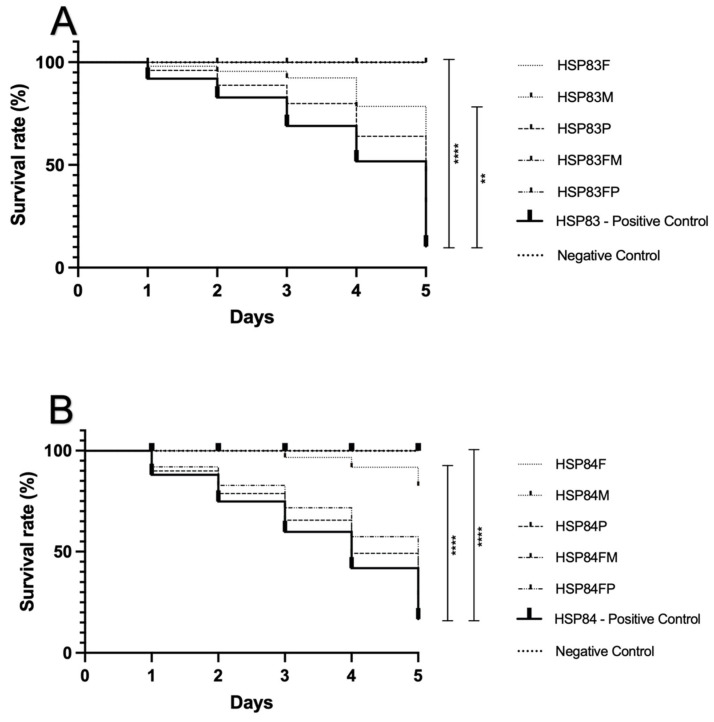
*Galleria mellonella* infection model. (**A**) Kaplan–Meier plots for larvae inoculated with HSP83 isolate. The curves obtained indicate that fosfomycin and meropenem alone, as well as the fosfomycin + meropenem combination, presented a significative higher survival rate compared to a positive control (bacteria inoculated and not treated). Polymyxin B in monotherapy and fosfomycin + polymyxin B combination did not achieve a significant difference. (**B**) Kaplan–Meier plots for larvae inoculated with HSP84 isolate. The curves obtained indicate that fosfomycin and meropenem alone, as well as fosfomycin in combination with meropenem or polymyxin B, presented a significative higher survival rate compared to a positive control (bacteria inoculated and not treated). Polymyxin B in monotherapy did not achieve a significant difference. F, fosfomycin alone; M, meropenem alone; P, polymyxin B alone; FM, fosfomycin + meropenem combination; FP, fosfomycin + polymyxin B combination; HSP83- or HSP84-positive control, larvae not treated; negative control, larvae inoculated with saline. (*p*-value: ** < 0.005; **** < 0.0001). The assay was performed with ten larvae per group and in duplicate.

Five days after larvae inoculation and treatment, we observed 100% larvae survival in the groups treated with fosfomycin + meropenem. Larvae inoculated with HSP83 isolate also presented survival rates of 100% when treated with fosfomycin + polymyxin B and fosfomycin alone. For those receiving meropenem alone and polymyxin B alone, survival rates were 75% and 55%, respectively. For larvae inoculated with HSP84 isolate, the survival rates were 50% for those treated with fosfomycin + polymyxin B, 90% for fosfomycin alone, 100% for meropenem alone, and 45% for polymyxin B alone (Figure 2).

The statistical analysis revealed that the curves obtained for HSP83 indicated that fosfomycin and meropenem alone, as well as fosfomycin in combination with meropenem, presented a significative higher survival rate compared to a positive control (bacteria inoculated and not treated; *p*-value < 0.0001). Polymyxin B in monotherapy and in combination with fosfomycin did not achieve a significant difference. For HSP84, the curves obtained indicated that fosfomycin and meropenem alone, as well as fosfomycin in combination with meropenem or polymyxin B, presented a significative higher survival rate compared to a positive control (bacteria inoculated and not treated) (*p*-values < 0.005 and <0.0001). Polymyxin B in monotherapy did not achieve a statistically significant difference.

### 2.4. Biofilm Assay

Among the 17 KPC-KPN, nine isolates were weakly adherent, eight were moderately adherent, and two were non-adherent isolates. The eight moderately adherent isolates (P16, P60, HSP65, HSP84, HSP83, P39, HSP29, and HSP64) were further selected for evaluation of the activity of antibiotics alone and in combination against biofilm formation. We observed a biofilm disruption in all isolates under exposure to antimicrobials alone and in combination (Table 5). The change in the ability of biofilm formation was observed from moderately to non-adherent in the presence of fosfomycin in combination with polymyxin B, fosfomycin alone, and polymyxin B alone (2.402–3.470-; 2.231–3.470-; 2.378–3.423-fold reduction on biofilm detection, respectively). For fosfomycin + meropenem and meropenem alone, six isolates changed to weakly adherent (1.481–2.724-; 1.335–2.385-fold reduction, respectively), and two isolates to non-adherent (2.016- and 2.357-; 2.052- and 2.489-fold reduction, respectively) (Table 5). In general, higher biofilm disruption was observed under the exposure to fosfomycin + polymyxin B, followed by polymyxin B and fosfomycin alone (*p* < 0.001).

## 3. Discussion

Difficult-to-treat infections caused by KPC-KPN are associated with high mortality rates. Even though novel therapeutic options such as the new β-lactamases-β-lactam inhibitor combinations and cefiderocol have become available for the treatment of KPC-producing Enterobacterales infections, access to these agents is still limited in low- and middle-income countries. Delays in the approval registration and cost have been impediments to the wider use of these new agents [3]. In this manner, old antimicrobial agents such as polymyxins and fosfomycin still constitute alternative therapeutic options to treat infections caused by MDR bacteria despite the risk of toxicity and emergence of resistance [9,23]. Despite its toxicity and increasing levels of resistance, polymyxin B still remains an alternative therapeutic option for treating KPC-KPN infections because it is widely available in Brazilian hospitals [24,25]. On the other hand, fosfomycin has high safety and displays low resistance rates [26,27]. The present study was performed with contemporaneous recovered Brazilian KPC-KPN isolates from different STs, and in the time–kill assay, we used peak and trough concentrations to observe if the evaluated combinations would be synergistic. In addition, the activity of these combinations was also in vivo assessed by testing the *G. mellonella* survival model.

In our study, fosfomycin showed a high susceptibility rate (76.5%) against XDR CRKP, with all harboring KPC and ESBL encoding genes. A similar susceptibility rate was encountered by Liu and collaborators, who reported high susceptibility rates for fosfomycin against CRKP (79.0%) or ESBL-producing *K. pneumoniae* (80.6%) [28]. In contrast, Al-Quraini et al. showed that XDR *K. pneumoniae* (MIC ≤ 32 mg/L) exhibited low fosfomycin susceptibility rates (33.3%; 5/15) [14]. Co-resistance to fosfomycin and meropenem or polymyxin B was found in four and three tested isolates, respectively.

Recently, Scudeller and collaborators performed a systematic review and meta-analysis and described high and moderate synergy rates of fosfomycin + polymyxins against CRKP [29]. Other studies also showed high rates of synergy for the fosfomycin + meropenem combination [14,16,30]. In the present study, by CKA synergistic effect was observed for fosfomycin + meropenem and fosfomycin + polymyxin B against 58.8% and 47.1% of the KPC-KPN isolates, respectively (Table 1 and Table 2). Furthermore, we observed meropenem and polymyxin B activity restoration with significant MIC fold decrease when meropenem or polymyxin B was combined with fosfomycin (Table 1). Our results corroborate the findings of a previous Brazilian study that tested KPC-KPN isolated before 2010. The authors showed that MIC_50_s and MIC_90_s were 32 and 256 mg/L for meropenem and 64 and 512 mg/L for fosfomycin, respectively. The antimicrobial combination increased bacterial susceptibility to 1/4 the MIC_50_s and to 1/8 to 1/16 the MIC_90_s of monotherapy. The antimicrobial combination demonstrated a synergistic effect for at least two-thirds of the isolates [30].

Among the STs evaluated in this study, STs 258 and ST437 presented higher rates of synergism for both combinations (fosfomycin + meropenem and fosfomycin + polymyxin B). This result might be extrapolated for other geographic regions because CC258 has been directly associated with *bla*_KPC-2_ dissemination worldwide [23]. The synergism rates were similar among KPC-KPN isolates independent of the ESBL co-production (Table 2). The CKA results were corroborated by the TKA findings that showed a high synergistic effect against KPC-2-producing isolates, especially at peak concentrations of combinations. Moreover, we could observe that four cases of partial synergism in CKA resulted in synergism in TKA for fosfomycin + polymyxin B (HSP29, HSP80, and HSP83) and fosfomycin + meropenem (P05), as displayed in Table 4.

Also, for the combination groups that we observed to be synergistic in CKA but not in TKA at 24 h, we observed that the synergistic effect occurred earlier and was lost as time went by. For example, the fosfomycin + meropenem combination was effective at 6 h at peak concentrations and trough concentrations for the isolate HSP29 and HSP06. We also observed a similar result for the fosfomycin + polymyxin B combination against HSP84. This combination was also effective at 6 h at peak concentrations. The lack of synergistic activity at 24 h was mainly due to bacterial regrowth observed for all isolates and combinations, except for fosfomycin + meropenem and fosfomycin + polymyxin B against HSP29 and HSP84, respectively. Our results are in line with previous reports of in vitro synergism assays with fosfomycin and polymyxin B. The regrowth 6 h after suggests the emergence of resistant isolates. This is one of the reasons why some specialists recommend avoiding the prescription of these antimicrobials in monotherapy to treat severe infections [15].

In order to evaluate if our in vitro results would be corroborated by those found in animal models, we performed a *G. mellonella* in vivo model to test fosfomycin combinations against KPC-2-producing *K. pneumoniae*. In the present study, the in vivo *G. mellonella* assay further confirmed the effectiveness of antimicrobial combinations, as shown in Figure 2. Synergism with fosfomycin in the *G. mellonella* model was previously reported, but to the best of our knowledge, there is no report in the literature testing fosfomycin alone and in combination to treat larvae infected with KPC-KPN. Kussmann and collaborators also reported high synergistic in vivo activity of fosfomycin combined with cefazolin resulting in a 44–54% reduction of larvae mortality infected with *Staphylococcus aureus,* which agreed with their in vitro data [31]. Thus, the concordance between the in vitro and in vivo (*G. mellonella)* results supports the use of this model to select the most appropriate therapeutic regimens [32,33,34]. Additionally, we observed fosfomycin bactericidal activity when used alone in the in vivo model (Figure 2), corroborating the in vitro data observed on TKA at 3 h at peak concentration (Table S1). However, the high activity of fosfomycin alone was observed in larvae five days after inoculation and treatment, suggesting that the immune system helped in combating the infection. Previously, it has been shown that fosfomycin has an immunomodulatory effect on cytokines production and NF-kB signaling pathway, modulating the function of B and T lymphocytes, monocytes, and neutrophils [35].

Finally, different studies have reported the fosfomycin activity on biofilm because this antimicrobial is capable of penetrating into these structures. Many studies have been performed with *Staphylococcus* spp., *Pseudomonas* spp., and *Escherichia coli* and show biofilm disruption with changes in biofilm structure leading to biofilm eradication [36,37,38,39,40]. In the present study, although we have observed biofilm disruption in all tested isolates under all the different exposures, greater activity was achieved by fosfomycin when combined with polymyxin B (*p* < 0.001) (Table 5 and Table S4). This interference on biofilm allowed the change in the isolate’s biofilm categorization from moderately adherent to weakly or non-adherent (majority). Recently, synergistic activity of fosfomycin combined with colistin or tigecycline against *K. pneumoniae* biofilms was reported [36,40]. Our findings are in agreement with these results. In addition, our study also showed the antibiofilm activity of fosfomycin combined with meropenem against *K. pneumoniae*, an exposure not previously investigated. This fosfomycin activity on biofilm in *K. pneumoniae* shows the promising use of this antimicrobial against different types of infection. *K. pneumoniae* is the second most common Gram-negative pathogen in osteomyelitis, and acute bacterial skin and skin structure infections, and these isolates are related to biofilm production. These types of infection are recognized to present high levels of biofilm formation, configuring difficult-to-treat infections [41,42]. Thus, fosfomycin (alone or in combination) could be a great alternative to antimicrobial therapy in these cases.

## 4. Materials and Methods

### 4.1. Bacterial Isolates

A total of 17 KPC-2-producing *K. pneumoniae* isolates previously characterized by whole-genome sequencing were studied [43]. These KPC-KPN isolates carried different resistance-encoding genes, with all isolates possessing chromosomally encoded *fosA.* In contrast, none harbored plasmid-encoded polymyxin resistance genes. All the isolates were classified as extensively drug-resistant (XDR) and presented different sequence types (ST): ST101 (*n* = 1), ST15 (*n* = 3), ST16 (*n* = 3), ST11 (*n* = 3), ST258 (*n* = 3), and ST437 (*n* = 4). Table S2 shows the complete resistome and the Genbank accession numbers of the isolates.

### 4.2. Antimicrobial Susceptibility Testing (AST)

The antimicrobial susceptibility profile of the *K. pneumoniae* isolates was determined by the agar dilution method for all antimicrobials tested, aztreonam, fosfomycin, imipenem, ertapenem, meropenem, amikacin, gentamycin, tigecycline, ciprofloxacin, levofloxacin, ceftazidime, and cefepime, except for polymyxin B, which was tested by broth microdilution following the BrCAST/EUCAST recommendations. The plates containing fosfomycin were supplemented with glucose-6-phosphate at 25 mg/L. Quality control and the interpretation of results were performed according to BrCAST/EUCAST guidelines, with results following within the expected ranges. We used as control the strains *Escherichia coli* ATCC 25922, *Pseudomonas aeruginosa* ATCC 27853, and *Staphylococcus aureus* ATCC 29213 [44,45].

### 4.3. Checkerboard Assay (CKA)

Synergy assessment by CKA was performed as previously described [46]. Sterile 96-well microdilution plates containing Mueller–Hinton broth with different concentrations of fosfomycin alone or in combination with meropenem and polymyxin B were used. A standard 0.5 McFarland inoculum containing approximately 1.5 × 10^8^ CFU/mL was added to each well, and the plates were incubated at 37 °C for 24 h. *K. pneumoniae* ATCC 70063 was tested as a quality control, and the experiments were performed in duplicate. The fractional inhibitory concentration summation (ΣFIC) index indicates the efficacy of the antimicrobial combinations used. ΣFIC is calculated as follows: ΣFIC = FIC A + FIC B, where FIC A is the MIC of drug A in combination/MIC of drug A alone, and FIC B is the MIC of drug B in combination/MIC of drug B alone. Classification of the antimicrobial effect of the combination was based on ΣFIC as follows: ΣFIC ≤ 0.5 indicates synergism; 0.5 < ΣFIC ≤ 1 indicates partial synergism; 1 < ΣFIC ≤ 2 indicates indifference; and ΣFIC > 4 indicates antagonism [46].

### 4.4. Time–Kill Assay (TKA)

Six representatives of KPC-2-producing *K. pneumoniae* isolates that presented synergism or partial synergism in CKA, harboring different resistance genes, presenting different phenotypes, and belonging to distinct STs were submitted to TKA. Isolates were selected as follows: HSP80, HSP84, HSP06, HSP29, HSP83, and P05. Table 1, Tables S2 and S3 summarize the isolates’ CKA results, resistome, and phenotype, respectively. Free peak and trough concentrations of fosfomycin, meropenem, and polymyxin B alone and in combination were used based on the clinical doses standardized by the EUCAST/BrCAST recommendations for intravenous regimens [44] as follows: fosfomycin 8 g q8h (peak, 395 mg/L; trough, 25 mg/L) [47], meropenem 2 g single dose (peak, 40.9 mg/L; trough, 4.3 mg/L) [48,49] and polymyxin B 25,000 U/kg/day (peak, 6.44 mg/L; trough, 2.4 mg/L) [50]. The viable colony counts were determined at 0, 3, 6, and 24 h. Synergism and antagonism were defined as a ≥ 2 − log_10_ CFU/mL decrease and increase, respectively, in the bacterial growth in combination when compared with the most active single agent at 24 h. Values between these ranges were considered as indifferent [46]. TKA was performed in duplicate. Briefly, a standard McFarland 0.5 inoculum was prepared using fresh colonies grown on MacConkey agar. Briefly, 100 μL of this inoculum was diluted in 5 mL of Muller Hinton Broth Cation Adjusted (CAMHB) and incubated at 37 °C until exponential growth (approximately 3 h). The inoculum was then adjusted to a McFarland 1.0 (~3 × 10^8^ CFU/mL) standard with sterile NaCl 0.9%. The final inoculum was prepared using 1 mL of the McFarland 1.0 inoculum plus 4 mL of CAMHB (~6 × 10^7^ CFU/mL). In each tube containing the antimicrobial to be tested as well as the combinations, 100 μL of inoculum (~6 × 10^7^ CFU/mL) were added. The positive control (tube with no drug) and negative control (sterility test) were included. Then, before the incubation at 37 °C, the first culture was performed (T = 0). All tubes containing fosfomycin were supplemented with glucose-6-phosphate at 25 mg/L. A serial six-dilution was performed from the initial inoculum, and then aliquots of 10 μL from each were plated in Muller Hinton agar plates. After 24 h of incubation, the colonies counting was performed.

### 4.5. Galleria Mellonella Selection, Inoculation, and Treatment

The *G. mellonella* model was tested as the infection animal model for the treatment of infections caused by two different *K. pneumoniae* clones harboring distinct resistance genes. HSP83 was susceptible to fosfomycin (MIC, 16 mg/L) and resistant to polymyxin B (MIC, 64 mg/L) and meropenem (MIC, 64 mg/L), and harbored *bla*_KPC-2_ and *bla*_SHV-182_. HSP84 was resistant to fosfomycin (MIC, 256 mg/L), polymyxin B (MIC, 64 mg/L), and meropenem (MIC, 64 mg/L) and harbored *bla*_KPC-2_, *bla_S_*_HV-182_, *bla*_TEM-1B_, *bla*_CTX-M-15_, and *bla*_OXA-1_ (Table 1). Healthy larvae weighing 230–280 mg were selected and administered first with 10 μL of the bacteria inoculum (1.5 × 10^8^ CFU/mL) and one hour later with the antimicrobial alone or in combination (10 μL) at peak doses. The inoculum was delivered in the last right proleg, and the treatment was injected in the last left proleg by using an insulin syringe. Ten larvae were included in each tested group, and the experiment was performed in duplicate. We used two control groups: one injected with saline (negative control) and the other one injected only with the bacterial inoculum (positive control). After the injection, larvae were incubated at 37 °C, and survival was observed daily for five consecutive days.

Before performing the in vivo animal model of infection, the larvae were inoculated only with the antimicrobial agents (fosfomycin, meropenem, polymyxin B) or their combinations, fosfomycin + meropenem or fosfomycin + polymyxin B, at peak concentrations to assess antimicrobial toxicity. The larvae were incubated at 37 °C, and survival rates were measured daily for five days.

### 4.6. Microtiter Plate Technique

The biofilm formation assay was performed using crystal violet on a polystyrene abiotic surface, and the results were interpreted as previously reported [51]. For those bacteria presenting moderate and strong adherence, these biofilms were further exposed to antimicrobials alone or in combination. Briefly, after 24 h at 37 °C of incubation, the medium was removed, and the wells were washed with PBS 1% two times. After this step, antimicrobial solution at peak concentrations was added to each well and incubated for more than 24 h at 37 °C. After incubation, the wells were washed three times with PBS 1% and fixed with formaldehyde 3%, and stained with crystal violet 1%. The dye was solubilized in ethanol 95%, and the OD was performed in a spectrophotometer at a wavelength of 570 nm. This assay was performed in triplicate.

### 4.7. Statistical Analysis

For *G. mellonella* analysis, the Kaplan–Meier survival curve was used, and the difference between the groups was determined by the log-rank (Mantel–Cox) test and Gehan–Breslow–Wilcoxon test. A *p*-value < 0.05 was considered statically significant. The analyses were performed in Prism 5.0 (GraphPad Prism Software, Inc., San Diego, CA, USA).

For the biofilm assay, we used the one-way ANOVA non-parametric (Kruskal–Wallis) test since the sample did not follow a normality pattern (Shapiro–Wilk test; W = 0.715; *p*-value < 0.001). In addition, we performed a comparison among the group tests using the Dwass–Steel–Critchlow–Fligner pairwise comparison. Test groups were not considered homogeneous by the Levine test (F = 17.5; *p*-value < 0.001). For this analysis, the Jamovi v. 1.6 software [52] was applied. This statistical analysis is displayed in Table S4.

## 5. Conclusions

Overall, we observed a high in vitro synergistic effect for both combinations, fosfomycin + meropenem and fosfomycin + polymyxin B, against CRKP isolates ST15, ST16, ST101, ST258, ST11, and ST437 despite the presence of multiple resistance genes. In addition, we could observe that when in combination with fosfomycin, meropenem, or polymyxin B presented, its susceptibility profile was restored in many isolates.

Our in vivo results are important because there is a worry about using fosfomycin (alone or in combination) to treat *K. pneumoniae* infections because of the regrowth observed in in vitro studies [15,53] and because it harbors an intrinsic *fosA* gene in its chromosome, which is usually not associated with resistance profile [54,55]. To date, just a single study reported fosfomycin resistance due to the expression of chromosomal *fosA* in *K. pneumoniae* [55].

We recognize that our work has limitations as the low number of isolates tested. Further studies testing a large number of isolates would be interesting. However, CKA and TKA are laborious and time-consuming. In addition, in our study, only isolates showing synergism or partial synergism in the CKA were selected further for TKA, which may be a cause of bias.

In conclusion, this work presents data on fosfomycin activity alone and in combination against 17 genetically characterized KPC-2-producing K. pneumoniae isolates through in vitro and in vivo assays, as well as its effect on biofilm formation. We hope that these data will help the clinician’s decisions on using intravenous fosfomycin in combination with the treatment of KPC-KPN infections.

## Figures and Tables

**Table 1:** CKA results and fosfomycin, β-lactams, and polymyxin resistance determinants for the isolates studied.

Isolate	ST	β-Lactamase Content	Polymyxin Resistance Determinants	*fosA*	MIC (mg/L) and MIC Category of Antimicrobials in Monotherapy				MIC (mg/L) and MIC Category of Antimicrobials in Combination										MIC Fold Decrease		ΣFIC/Interpretation	
					FOS		MEM		POLB		FOS + MEM				FOS + POL				FOS	FOS	FOS	FOS
											FOS		MEM		FOS		POLB		+	+	+	+
					MIC	C	MIC	C	MIC	C	MIC	C	MIC	C	MIC	C	MIC	C	MEM	POLB	MEM	POL
P60	ST101	*bla*_KPC-2_; *bla*_SHV-28_; *bla*_TEM-1B_; *bla*_CTX-M-15_		+	16	S	128	R	2	S	8	S	**0.25**	**S**	8	S	0.5	S	2; 512	2; 4	0.5/ SYN	0.75/PSYN
HSP29	ST11	*bla*_KPC-2_; *bla*_SHV-182_		+	64	R	128	R	1	S	**4**	**S**	**0.125**	**S**	**4**	**S**	0.5	S	16; 1024	16; 2	0.06/ SYN	0.53/PSYN
HSP64	ST11	*bla*_KPC-2_; *bla*_SHV-182_; *bla*_TEM-1B_	Deletion in *mgrB* (aa46)*;* mutation in *crrB* (D189E)*; pmrB* (R256G); mutation in *pmrC* (Q319R)	+	16	S	128	R	8	R	32	S	**1**	**S**	64	R	**0.5**	**S**	0.5; 128	0.25; 16	2.01/ IND	2.03/ IND
HSP73	ST11	*bla*_KPC-2_; *bla*_SHV-182_	*mgrB* disruption; mutation in *crrB* (D189E); *pmrB* (R256G); mutation in *pmrC* (Q319R)	+	8	S	64	R	8	R	4	S	4	I	8	S	**0.5**	**S**	2; 16	1; 16	0.56/PSYN	1.06/ IND
P16	ST15	*bla*_KPC-2_; *bla*_SHV-28_; *bla*_CTX-M-15_		+	16	S	32	R	2	S	16	S	**0.125**	**S**	4	S	0.25	S	1; 256	4; 8	1.0/IND	0.375/SYN
P35	ST15	*bla*_KPC-2_; *bla*_SHV-28_; *bla*_SHV-98_; *bla*_TEM-1B_; *bla*_CTX-M-15_	Mutation in *pmrB* (A246T)*;* mutation in *pmrC* (F27C; S257L)	+	16	S	64	R	16	R	1	S	4	I	4	S	**2**	**S**	16; 16	4; 8	0.13/SYN	0.375/SYN
P51	ST15	*bla*_KPC-2_; *bla*_SHV-28_; *bla*_CTX-M-15_	Mutation in *pmrB* (A246T)*;* mutation in *pmrC* (F27C; S257L)	+	4	S	32	R	16	R	2	S	4	I	4	S	**2**	**S**	2; 8	1; 8	0.63/PSYN	1.125/IND
HSP80	ST16	*bla*_KPC-2_; *bla*_SHV-1_; *bla*_TEM-1A_; *bla*_CTX-M-15_; *bla*_OXA-1_	Deletion in *mgrB* (aa3)*;* mutation in *crrB* (D189E; V237I)*;* mutation in *pmrC* (F27C)	+	256	R	128	R	32	R	**32**	**S**	**0.06**	**S**	128	R	8	R	8; 2048	2; 4	0.13/ SYN	0.75/PSYN
HSP83	ST16	*bla*_KPC-2_; *bla*_SHV-182_	*mgrB* disruption; mutation in *crrB* (D189E; V237I); *pmrB* (R256G); mutation in *pmrC* (Q319R)	+	16	S	64	R	64	R	4	S	**0.25**	**S**	8	S	**0.5**	**S**	4; 256	2; 128	0.25/ SYN	0.508/PSYN
P05	ST16	*bla*_KPC-2_; *bla*_SHV-1_; *bla*_TEM-1B_; *bla*_CTX-M-14_	Mutation in *crrB* (D189E; V237I); mutation in *pmrA* (E57G); mutation in *pmrC* (F27C)	+	16	S	16	R	16	R	16	S	**0.06**	**S**	4	S	**0.125**	**S**	1; 256	4; 128	1/PSYN	0.258/SYN
P86	ST258	*bla*_KPC-2_; *bla*_SHV-182_; *bla*_TEM-1B_; *bla*_CTX-M-14_	Mutation in *mgrB* (W36S), *crrB* (D189E; V237I; Q296L)*; pmrB* (R256G)*;* mutation in *pmrC* (Q319R)	+	16	S	4	I	16	R	1	S	**0.5**	**S**	4	S	4	R	16; 8	4; 4	0.19/SYN	0.50/SYN
P39	ST258	*bla*_KPC-2_; *bla*_SHV-182_; *bla*_TEM-1B_; *bla*_CTX-M-14_	Mutation in *crrB* (D189E; V237I; Q296L)*; pmrB* (R256G)*;* mutation in *pmrC (Q319R)*	+	16	S	64	R	4	R	8	S	**0.25**	**S**	8	S	**0.25**	**S**	2; 256	2; 16	0.5/SYN	0.56/PSYN
P71	ST258	*bla*_KPC-2_; *bla*_SHV-182_; *bla*_TEM-1B_; *bla*_CTX-M-14_	Mutation in *mgrB* (W36S), *crrB* (D189E; V237I; Q296L)*; pmrB* (R256G)*;* mutation in *pmrC* (Q319R)	+	8	S	4	I	4	R	0.5	S	**0.25**	**S**	2	S	**1**	**S**	16; 16	4; 4	0.13/SYN	0.5/SYN
HSP06	ST437	*bla*_KPC-2_; *bla*_SHV-182_; *bla*_OXA-1_		+	128	R	128	R	2	S	**8**	**S**	**0.25**	**S**	**16**	**S**	0.5	S	16; 512	8; 4	0.03/SYN	0.375/SYN
HSP17	ST437	*bla*_KPC-2_; *bla*_SHV-182_; *bla*_TEM-1B_; *bla*_CTX-M-15_	Mutation in *pmrB* (R256G)*;* mutation in *pmrC (Q319R)*	+	16	S	128	R	32	R	16	S	**0.5**	**S**	16	S	8	R	1; 256	1; 4	1.00/PSYN	1.25/IND
HSP84	ST437	*bla*_KPC-2_; *bla_S_*_HV-182_; *bla*_TEM-1B_; *bla*_CTX-M-15_; *bla*_OXA-1_	Mutation in *pmrB* (R256G)*;* mutation in *pmrC (Q319R)*	+	256	R	64	R	64	R	64	R	**0.06**	**S**	**32**	**S**	**0.5**	**S**	4; 1024	8; 128	0.25/SYN	0.13/SYN
P29	ST437	*bla*_KPC-2_; *bla*_SHV-182_; *bla*_TEM-1B_; *bla*_CTX-M-15_; *bla*_OXA-1_		+	16	S	64	R	2	S	8	S	**0.5**	**S**	4	S	0.5	S	2; 128	4; 4	0.51/PSYN	0.5/SYN
MIC_50_					16		64		8		8		0.25		8		0.5					
MIC_90_					256		128		64		32		4		64		8					

The MICs in combination, which changed the classification from resistant, R, or susceptible, increasing the exposure, I, to susceptible, S, are highlighted in bold. FOS, fosfomycin; MEM, meropenem; POLB polymyxin; C, category; SYN, synergism; PSYN, partial synergism; IND, indifferent.

**Table 2:** CKA results split by β-lactamase content.

β-Lactamases Genes	FOS + MEM			FOS + POL		
	Synergy, *n* (%)	Partial Synergy, *n* (%)	Indifference, *n* (%)	Synergy, *n* (%)	Partial Synergy, *n* (%)	Indifference, *n* (%)
*bla*_KPC-2_ (*n* = 17)	10 (58.8)	5 (29.4)	2 (11.8)	8 (47.1)	5 (29.4)	4 (23.5)
*bla*_KPC-2_ + *bla*_CTX-M-15_ + *bla*_OXA-1_ (*n* = 3)	2 (66.6)	1 (33.3)	0	2 (66.6)	1 (33.3)	0
*bla*_KPC-2_ + *bla*_CTX-M-15_ (*n* = 5)	2 (40.0)	2 (40.0)	1 (20.0)	2 (40.0)	1 (20.0)	2 (40.0)
*bla*_KPC-2_ + *bla*_CTX-M-14_ (*n* = 4)	3 (75.0)	1 (25.0)	0	3 (75.0)	1 (25.0)	0
*bla*_KPC-2_ + *bla*_OXA-1_ (*n* = 1)	1 (100)	0	0	1 (100)	0	0
No ESBL (*n* = 4)	2 (50)	1 (25)	1 (25)	0	2 (50)	2 (50)

FOS, fosfomycin; MEM, meropenem; POL, polymyxin.

**Table 3:** TKA results showing the growth rates for six representative isolates between time zero and 24 h.

	Change from Time Zero to 24 h (log_10_ CFU/mL)					
	HSP80	HSP84	HSP83	P05	HSP29	HSP06
FOS peak	0.56	2.38	−3.78	1.27	−6.60	2.71
FOS trough	3.04	2,46	2.90	2.93	3.09	3.24
MEM peak	2.66	3.37	2.60	3.06	−6.00	3.74
FOS + MEM peak	**−3.78**	**−3.00**	**−6.50**	**−6.00**	−6.00	1.40
MEM trough	3.32	3.74	2.95	2.99	0.84	3.24
FOS + MEM trough	2.78	3.27	3.43	3.27	2.84	3.74
POL peak	1.70	−6.50	−1.98	1.53	−6.00	2.51
FOS + POL peak	**−6.21**	−6.23	**−6.00**	**−6.14**	−6.00	**−6.00**
POL trough	3.24	3.02	1.72	2.08	1.74	2.05
FOS + POL trough	3.74	3.13	3.36	2.39	**−6.00**	0.36

The values indicate the change in log_10_ CFU/mL for each isolate, comparing the time zero and the time 24 h. Synergism was highlighted in bold. Synergism was defined as a ≥2 − log_10_ CFU/mL decrease in bacterial growth in combination when compared with the most active single agent at 24 h. FOS, fosfomycin; MEM, meropenem; POL, polymyxin.

**Table 4:** Comparison between CKA and TKA.

Isolate	Sequence Type	β-Lactamase Content	FOS + MEM	FOS + POLB	FOS + MEM	FOS + POLB
HSP 29	ST11	*bla*_KPC-2_; *bla*_SHV-182_	0.06/SYN	0.53/PSYN	-	SYN/trough
HSP 80	ST16	*bla*_KPC-2_; *bla*_SHV-1_; *bla*_TEM-1A_; *bla*_CTX-M-15_; *bla*_OXA-1_	0.13/SYN	0.75/PSYN	SYN/peak	SYN/peak
HSP 83	ST16	*bla*_KPC-2_; *bla*_SHV-182_	0.25/SYN	0.508/PSYN	SYN/peak	SYN/peak
P05	ST16	*bla*_KPC-2_; *bla*_SHV-1_; *bla*_TEM-1B_; *bla*_CTX-M-14_	1/PSYN	0.258/SYN	SYN/peak	SYN/peak
HSP 06	ST437	*bla*_KPC-2_; *bla*_SHV-182_; *bla*_OXA-1_	0.03/SYN	0.375/SYN	-	SYN/peak
HSP 84	ST437	*bla*_KPC-2_; *bla*_SHV-182_; *bla*_TEM-1B_; *bla*_CTX-M-15_; *bla*_OXA-1_	0.25/SYN	0.13/SYN	SYN/peak	-

FOS, fosfomycin; MEM, meropenem; POLB, polymyxin; SYN, synergism; PSYN, partial synergism; IND, indifferent.

**Table 5:** Biofilm assay results showing the optical densities values for each isolate at each different exposure to antimicrobials and combinations.

ISOLATE	NO DRUG	Classification	FOS	Classification	Biofilm Fold Reduction	FOS + MEM	Classification	Biofilm Fold Reduction	POLB	Classification	Biofilm Fold Reduction	FOS + POLB	Classification	Biofilm Fold Reduction	MEM	Classification	Biofilm Fold Reduction
P16	0.124	M	0.054	NA	2.313	0.078	W	1.604	0.048	NA	2.610	0.048	NA	2.574	0.093	W	1.335
P60	0.167	M	0.048	NA	3.470	0.061	W	2.724	0.049	NA	3.423	0.048	NA	3.470	0.070	W	2.385
HSP65	0.117	M	0.049	NA	2.397	0.075	W	1.575	0.048	NA	2.455	0.049	NA	2.405	0.064	W	1.830
HSP84	0.127	M	0.048	NA	2.633	0.054	NA	2.357	0.047	NA	2.681	0.048	NA	2.626	0.051	NA	2.489
HSP83	0.116	M	0.052	NA	2.231	0.072	W	1.611	0.048	NA	2.442	0.048	NA	2.408	0.080	W	1.453
HSP29	0.115	M	0.050	NA	2.298	0.057	NA	2.016	0.048	NA	2.378	0.048	NA	2.402	0.056	NA	2.052
HSP64	0.132	M	0.052	NA	2.516	0.089	W	1.481	0.048	NA	2.728	0.048	NA	2.728	0.098	W	1.350
P39	0.124	M	0.049	NA	2.551	0.059	W	2.092	0.047	NA	2.667	0.049	NA	2.522	0.058	W	2.132

Values indicate the optical density obtained in the spectrophotometer. FOS, fosfomycin; MEM, meropenem; POLB, polymyxin; M, moderately adherent; W, weakly adherent; NA, non-adherent.

## Data Availability

Not applicable.

## Supplementary Materials

The following supporting information can be downloaded at: https://www.mdpi.com/article/10.3390/antibiotics12020237/s1, Table S1: TKA results in log_10_ CFU/mL for each isolate in each different time and at different concentrations; Table S2: Complementary information about the isolates.; Table S3: Antimicrobial susceptibility testing; Table S4: Statistical analysis for biofilm disruption under antimicrobials combination exposure assay.
