# Supplementary material for: In Vitro and In Vivo Synergism of Fosfomycin in Combination with Meropenem or Polymyxin B against KPC-2-Producing Klebsiella pneumoniae Clinical Isolates
Source: Antibiotics (Basel). 2023 Jan 23;12(2):237. doi: 10.3390/antibiotics12020237 (PMC9952190; doi:10.3390/antibiotics12020237)
Supplement: Supplementary file 1 [file antibiotics-12-00237-s001.zip › antibiotics-2168058-supplementary.pdf]

Table S1: TKA results in log_{10} CFU/mL for each strain in each different time and at different concentrations.

	HSP80	HSP84	HSP83	P05	HSP29	HSP06	Mean	Mean decrease/increase
**Control**								
T=0	6,70	6,50	6,70	6,60	6,50	6,19	6,53	
T=3h	9,74	8,28	8,74	9,26	9,17	9,74	9,16	2,63
T=6h	9,74	9,74	9,20	9,74	9,74	9,74	9,65	3,12
T=24h	9,74	9,74	9,74	9,17	9,74	9,74	9,65	3,12
**FOS peak**								
T=0	6,50	5,99	6,30	6,60	6,68	6,57	6,44	
T=3h	3,93	3,06	2,40	3,00	2,52	3,82	3,12	-3,32
T=6h	5,70	3,64	7,87	8,89	9,59	3,10	6,46	0,02
T=24h	7,06	8,37	2,52	7,87	0,00	9,28	5,85	-0,59
**FOS trough**								
T=0	6,71	6,50	6,50	6,20	6,65	6,50	6,51	
T=3h	7,46	9,74	7,39	8,92	6,22	9,21	8,16	1,65
T=6h	8,54	9,26	7,92	7,76	9,74	8,66	8,65	2,14
T=24h	9,74	9,74	9,40	9,13	9,74	9,74	9,58	3,07
**MERO peak**								
T=0	6,50	5,80	6,50	6,40	6,00	6,00	6,20	
T=3h	4,90	5,79	7,50	8,28	2,96	3,03	5,41	-0,79
T=6h	7,88	4,29	8,31	9,53	2,22	4,69	6,15	-0,05
T=24h	9,16	9,17	9,10	9,46	0,00	9,74	7,77	1,57
**MERO + FOS peak**								
T=0	6,00	6,00	6,67	6,00	6,00	6,00	6,11	
T=3h	3,00	4,04	0,00	0,00	0,00	1,92	1,49	-4,62

T=6h	2,96	3,30	0,00	0,00	0,00	0,00	1,04	-5,07
T=24h	2,22	3,00	0,00	0,00	0,00	7,40	2,10	-4,01
**MERO trough**								
T=0	6,42	6,00	6,45	6,50	6,34	6,50	6,37	
T=3h	8,66	8,90	9,74	9,20	3,61	8,68	8,13	1,76
T=6h	9,74	9,74	9,27	9,74	9,74	9,67	9,65	3,28
T=24h	9,74	9,74	9,40	9,49	7,19	9,74	9,22	2,85
**MERO + FOS trough**								
T=0	6,50	6,00	6,00	6,00	6,00	6,00	6,08	
T=3h	8,49	8,22	4,16	5,46	3,20	9,49	6,50	0,42
T=6h	9,41	9,02	8,53	8,11	1,92	7,25	7,37	1,29
T=24h	9,28	9,27	9,43	9,27	6,04	9,74	8,84	2,76
**POLI peak**								
T=0	6,55	6,50	6,20	5,82	6,00	6,00	6,18	
T=3h	5,18	7,34	3,28	6,59	0,00	0,00	3,73	-2,45
T=6h	2,69	3,64	1,92	7,02	0,00	0,00	2,55	-3,63
T=24h	8,25	0,00	4,22	7,35	0,00	8,51	4,72	-1,46
**POLB + FOS peak**								
T=0	6,21	6,23	6,00	6,14	6,00	6,00	6,10	
T=3h	4,01	3,00	0,00	0,00	0,00	0,00	1,17	-4,93
T=6h	0,00	0,00	0,00	0,00	0,00	0,00	0,00	-6,10
T=24h	0,00	0,00	0,00	0,00	0,00	0,00	0,00	-6,10
**POLB trough**								
T=0	6,50	6,40	6,63	6,50	6,00	6,00	6,34	
T=3h	8,94	9,21	7,80	9,13	0,00	0,00	5,85	-0,49
T=6h	9,74	9,48	8,39	8,82	6,19	0,00	7,10	0,77

T=24h	9,74	9,42	8,34	8,58	7,74	8,05	8,65	2,31
**POLB + FOS trough**								
T=0	6,00	6,00	6,00	6,10	6,00	6,00	6,02	
T=3h	9,74	9,14	5,08	8,23	0,00	7,83	6,67	0,65
T=6h	9,74	9,37	8,48	8,59	0,00	1,92	6,35	0,33
T=24h	9,74	9,13	9,36	8,49	0,00	6,36	7,18	1,17

Table S2: Complementary information about the strains.

		Antimicrobial class – Resistance genes								
**Strain**	**ST**	**Fosfomycin**	**β-lactams**	**Quinolones**	**Macrolides**	**Tetraciclyne**	**Sulfonamides**	**Aminoglycosides**	**Fenicol**	**Trimethoprim**	**Accesion number**
P60	ST101	*fosA*	*bla*$_{KPC-2}$, *bla*$_{SHV-28}$, *bla*$_{TEM-1B}$, *bla*$_{CTX-M-15}$	*oqxA*			sul2	*aac(6')-Ib3* *aph(3')-Ia*	*catB3*	*dfrA14*	SAMEA7556476
HSP29	ST11	*fosA*	*bla*$_{KPC-2}$, *bla*$_{SHV-182}$	*oqxA, oqxB*		*tet(A)*				*dfrA30*	SAMEA7556444
HSP73	ST11	*fosA*	*bla*$_{KPC-2}$, *bla*$_{SHV-182}$	oqxB		*tet(A)*		*aph(3')-Via*		*dfrA30*	SAMN10592697
HSP64	ST11	*fosA*	*bla*$_{KPC-2}$, *bla*$_{SHV-182}$, *bla*$_{TEM-1B}$	*oqxA, oqxB, aac(6')-Ib-cr*		*tet(A)*	*sul1*	*aac(6')-Ib3, aac(3)-Iia*	*catA1*	*dfrA30*	SAMEA7556448
HSP06	ST437	*fosA*	*bla*$_{KPC-2}$, *bla*$_{SHV-182}$, *bla*$_{CTX-M-14}$, *bla*$_{OXA-1}$	*oqxA, oqxB*	*mph(A)*		*sul1*	*aac(6')-Ib3, aph(3')-Ia*	*catB3*	*dfrA30*	SAMN10592704
HSP84	ST437	*fosA*	*bla*$_{KPC-2}$, *bla*$_{SHV-182}$, *bla*$_{TEM-1B}$, *bla*$_{CTX-M-15}$, *bla*$_{OXA-1}$	*oqxA, oqxB, aac(6')-Ib-cr*	*mph(A)*	*tet(A), tet(D)*	*sul1*	*aac(3')-Iid, aph(3')-Ia*	*catB3*	*dfrA30*	SAMEA7556454
P29	ST437	*fosA*	*bla*$_{KPC-2}$, *bla*$_{SHV-182}$, *bla*$_{TEM-1B}$, *bla*$_{CTX-M-15}$, *bla*$_{OXA-1}$	oqxB		*tet(A), tet(D)*		*aph(3')-Ia*		*dfrA30*	SAMN10592700

HSP17	ST437	*fosA*	*bla*$_{\text{KPC-2}}$, *bla*$_{\text{SHV-182}}$, *bla*$_{\text{TEM-1B}}$, *bla*$_{\text{CTX-M-15}}$	*oqxA, oqxB, aac(6’)-Ib-cr*	*mph(A)*	*tet(A), tet(D)*		*aac(3’)-Iid, aph(3’)-Ia*	*catB3*	*dfrA30*	SAMN10592705
P16	ST15	*fosA*	*bla*$_{\text{KPC-2}}$, *bla*$_{\text{SHV-28}}$, *bla*$_{\text{CTX-M-15}}$	oqxB, qnrB1	*mph(A)*		*sul1*		*catA1*	*dfrA12*	SAMN14770896
P51	ST15	*fosA*	*bla*$_{\text{KPC-2}}$, *bla*$_{\text{SHV-28}}$, *bla*$_{\text{CTX-M-163}}$	oqxB, aac(6’)-Ib-cr, qnrB1	*mph(A)*		sul2	*aac(6’)-Ib-cr*	*catA1*		SAMN14763866
P35	ST15	*fosA*	*bla*$_{\text{KPC-2}}$, *bla*$_{\text{SHV-28}}$, *bla*$_{\text{SHV-98}}$, *bla*$_{\text{TEM-1B}}$, *bla*$_{\text{CTX-M-15}}$	*oqxA, oqxB, qnrB1*	*mph(A)*		sul2		*catA1*		SAMN14770898
HSP83	ST16	*fosA*	*bla*$_{\text{KPC-2}}$, *bla*$_{\text{SHV-182}}$	*oqxA, oqxB, aac(6’)-Ib-cr*		*tet(A)*		*aph(3’)-Via*		*dfrA30*	ASM1103817v1
P05	ST16	*fosA*	*bla*$_{\text{KPC-2}}$, *bla*$_{\text{SHV-1}}$, *bla*$_{\text{TEM-1B}}$, *bla*$_{\text{CTX-M-14}}$	*oqxA, oqxB*	erm(42	tet(G)	*sul1, sul2*	*aac(3’)-Iid, aph(3’’’)-Ib, aph(6)-Id, rmtb*		*dfrA12*	ASM1103836v1
HSP80	ST16	*fosA*	*bla*$_{\text{KPC-2}}$, *bla*$_{\text{SHV-1}}$, *bla*$_{\text{TEM-1a}}$, *bla*$_{\text{CTX-M-15}}$, *bla*$_{\text{OXA-1}}$	oqxB	*mph(A)*			*aph(3’)-Ia*		*dfrA12*	ASM1103821v1
P39	ST258	*fosA*	*bla*$_{\text{KPC-2}}$, *bla*$_{\text{SHV-182}}$, *bla*$_{\text{TEM-1B}}$, *bla*$_{\text{CTX-M-14}}$	oqxB	*mph(A)*	tet(G)		*aac(3’)-Iid, aph(3’’’)-Ib*	*catA1*	*dfrA12*	SAMN10592671
P71	ST258	*fosA*	*bla*$_{\text{KPC-2}}$, *bla*$_{\text{SHV-182}}$, *bla*$_{\text{TEM-1B}}$, *bla*$_{\text{CTX-M-14}}$	oqxB		tet(G)	sul2	*aac(3’)-Iid, aph(3’)-Ia*	*catA1*		SAMN10592674
P86	ST258	*fosA*	*bla*$_{\text{KPC-2}}$, *bla*$_{\text{SHV-182}}$, *bla*$_{\text{TEM-1B}}$, *bl*$_{\text{aCTX-M-14}}$	*oqxA, oqxB*	*mph(A)*	tet(G)	sul2	*aac(3’)-Iid*	*catA1*	*dfrA12*	SAMEA7556485

Table S3: Antimicrobial Susceptibility Testing

**Strain**	**Antimicrobial agent**													
	AZT	IMI	MEM	ERT	AMK	GEN	TGC	FOS	CIP	LEV	CAZ	CRO	FEP	POLI
P60	**>64**	**8**	**128**	**64**	4	**>64**	1	16	**>64**	**64**	**128**	**>256**	**>256**	2
P35	**>64**	**128**	**64**	**16**	8	**64**	2	16	**>64**	**16**	**128**	**256**	**128**	**16**
HSP29	**>64**	**64**	**256**	**64**	8	**4**	4	**64**	**>64**	**32**	**128**	**256**	**128**	1
HSP17	**>64**	**64**	**128**	**64**	8	**>64**	2	16	**>64**	**16**	**128**	**>256**	**>256**	**32**
HSP06	**>64**	**64**	**128**	**64**	**>64**	**>64**	2	**128**	**>64**	**16**	**128**	**>256**	**>256**	2
P71	**>64**	4	4	**16**	**>64**	**>64**	1	8	**>64**	**32**	**128**	**256**	**>256**	4
P05	**>64**	**32**	**16**	**128**	**>64**	**>64**	2	16	**>64**	**16**	**256**	**>256**	**>256**	**16**
P16	**>64**	**64**	**32**	**256**	**64**	**64**	16	16	**>64**	>64	**128**	**>256**	**128**	2
P86	**64**	**8**	4	**128**	8	**4**	4	16	**>64**	**32**	**128**	**>256**	**>256**	**16**
P51	**>64**	**16**	**32**	**64**	**>64**	**>64**	4	4	**>64**	**16**	**128**	**>256**	**>256**	**16**
HSP84	**>64**	**32**	**64**	**64**	**>64**	**>64**	2	**256**	**>64**	**16**	**128**	**>256**	**>256**	**64**
HSP83	**>64**	**64**	**64**	>256	4	**>64**	2	16	**>64**	**32**	**256**	**>256**	**>256**	**64**
P29	**>64**	**32**	**64**	**256**	**16**	**>64**	4	16	**>64**	**16**	>256	**>256**	**>256**	2
HSP73	**>64**	**16**	**64**	**256**	**>64**	**>64**	8	8	**>64**	**16**	**256**	**>256**	**128**	**8**
P39	**>64**	**256**	**64**	**256**	**32**	**>64**	16	16	**>64**	>64	**128**	**>256**	**>256**	4
HSP80	**>64**	**256**	**128**	>256	**64**	**>64**	16	**256**	**>64**	>64	**256**	**>256**	**>256**	**32**
HSP64	**>64**	**256**	**128**	>256	**32**	**>64**	16	16	**>64**	**256**	**128**	**>256**	**128**	**8**

AZT, aztreonam; IMI, imipenem; MEM, meropenem; ERT, ertapenem; AMK, amikacin; GEN, gentamycin; TGC, tigeciclyne; FOS, fosfomycin; CIP, ciprofloxacin; LEV, levofloxacin; CAZ, ceftazidime; CRO, ceftriaxone; FEP, cefepime; POLB, polymyxin B.

Table S4: Statistical analysis for biofilm disruption under antimicrobials combination exposure assay.

Biofilm disruption statistical Analysis

Normality Test (Shapiro-Wilk)

	W	p
OD	0.715	< .001

Homogeneity of Variances Test (Levene's)

	F	df1	df2	p
OD	17.5	5	282	< .001

One-Way ANOVA (Non-parametric) - Kruskal-Wallis

	χ^2	df	p
OD	221	5	< .001

Dwass-Steel-Critchlow-Fligner pairwise comparisons

Pairwise comparisons - OD

Standard group	Comparison group	W	p
FOS	FOS+MEM	8.834	< .001
FOS	FOS+POL	-4.429	0.022
FOS	MEM	9.001	< .001
FOS	NO DRUG	11.857	< .001
FOS	POL	-5.566	0.001
FOS+MEM	FOS+POL	-11.613	< .001
FOS+MEM	MEM	0.706	0.996
FOS+MEM	NO DRUG	11.424	< .001
FOS+MEM	POL	-11.725	< .001
FOS+POL	MEM	11.622	< .001
FOS+POL	NO DRUG	11.978	< .001

FOS+POL	POL	-1.761	0.815
MEM	NO DRUG	10.800	< .001
MEM	POL	-11.697	< .001
NO DRUG	POL	-11.959	< .001
